# Cloning, Expression, and *in vitro* Functional Activity Assay of phiC31 Integrase cDNA in Escherichia coli

**Published:** 2013-02-20

**Authors:** Mohammad Hadi Sekhavati, Mojtaba Tahmoorespur, Kamran Ghaedi, Kianoush Dormiani, Mohammad Reza Nassiri, Yahya Khazaie, Mahboubeh Foruzanfar, Morteza Hosseini, Mohammad Hossein Nasr Esfahani

**Affiliations:** 1. Department of Animal Science, Ferdowsi University of Mashhad, Mashhad, Iran; 2. Department of Molecular Biotechnology at Cell Science Research Center, Royan Institute for Biotechnology, ACECR, Isfahan, Iran; 3. Department of Biology, School of Sciences, University of Isfahan, Isfahan, Iran; 4. Department of Pharmaceutical Biotechnology, Faculty of Pharmacy and Pharmaceutical Sciences, Isfahan University of Medical Sciences, Isfahan, Iran; 5. Department of Reproductive Biotechnology at Reproductive Biomedicine Research Center, Royan Institute for Biotechnology, ACECR, Isfahan, Iran

**Keywords:** phiC31, Site-Specific Integration, *E.coli* BL21 (DE3)

## Abstract

**Objective:**

The aim of present study was cloning and expression of phiC31 integrase cDNA in a bacterial expression vector. Thus, an intra molecular assay vector was applied to show *in vitro* activity of recombinant protein.

**Materials and Methods:**

In this experimental study, phiC31 cDNA was subcloned into a prokaryotic expression vector and transformed into *E.coli* Bl21 (DE3). Recombinant phiC31 integrase was purified form the bacterial cell lysates and its activity was verified by an *in vitro* functional assessment.

**Results:**

Sodium dodecyl sulfate-polyacrylamide gel electrophoresis (SDS-PAGE) of the puriﬁed phiC31 integrase confirmed the size of protein (70 kDa). Finally, the functionality of purified phiC31 integrase was verified.

**Conclusion:**

The results of this study indicated that the purified integrase has a great potential application for *in vitro* site-specific integration.

## Introduction

phiC31 integrase is a site-specific recombinase, which is produced by a kind of bacteriophage (phiC31) in a strain of Streptomyces (1, 2). This enzyme is a member of serine recombinases that has an accurate and specific function to recombine DNA fragments between two identified sites, with approximately 30 base pairs (3). In nature, these two sites, *attP* and *attB*, are present in the phage and its host (*Streptomyces*) genomes, respectively (1). phiC31 integrase has a well ability to recombine these two sites and generate the hybrid *attL* and *attR* sites, which cannot be reacted by this enzyme, inversely. Moreover, phiC31 integrase is not required to have any host-specific co-factors for its activity. These two unique features of phiC31 integrase among other recombinases make it appropriate for *in vitro* site-specific recombination (4). On the other hand, several studies have reported that there are some site-specific regions in the mammalian genomes similar to *attP*, which are known as pseudo *attP* (5, 6, 7). These reports encouraged researchers to investigate the use of phiC31 integrase as a tool in human gene therapy and creation of transgenic animals (5, 8, 9).

The integrase open reading frame encoding phiC31 integrase protein was previously identified by Kuhstoss and Rao in 1991 (1). Functional protein, has two main domains, including the N-terminal (NTD) and C-terminal domain (CTD) (10). The NTD involves in catalytic activity for DNA cleavage, strand exchange and joining of recombination products (11, 12), while CTD is responsible for DNA binding, controlling of integration, and excision (13, 14).

One of the unique features of phiC31 integrase is its ability for *in vitro* site-specific recombination. Thorpe and Smith showed *in vitro* using of phiC31 integrase could combine two plasmids, containing att sites, which generated one recombinant plasmid (4). Therefore, it seems that recombinant phiC31 integrase could be useful for further approaches in genetic engineering for site-specific recombination. In this study, we produced and purified the expressed phiC31 integrase from *E.coli* BL21 (DE3). Then *in vitro* activity of recombinant enzyme was determined.

## Materials and Methods

### Plasmids

**Fig 1:**
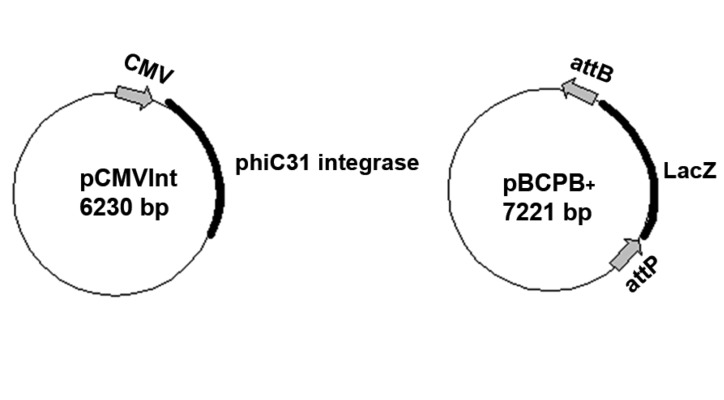
Schematic diagrams of two plasmids used for phiC31 integrase expression and *in vitro* phiC31 integrase activity.

**Fig 2:**
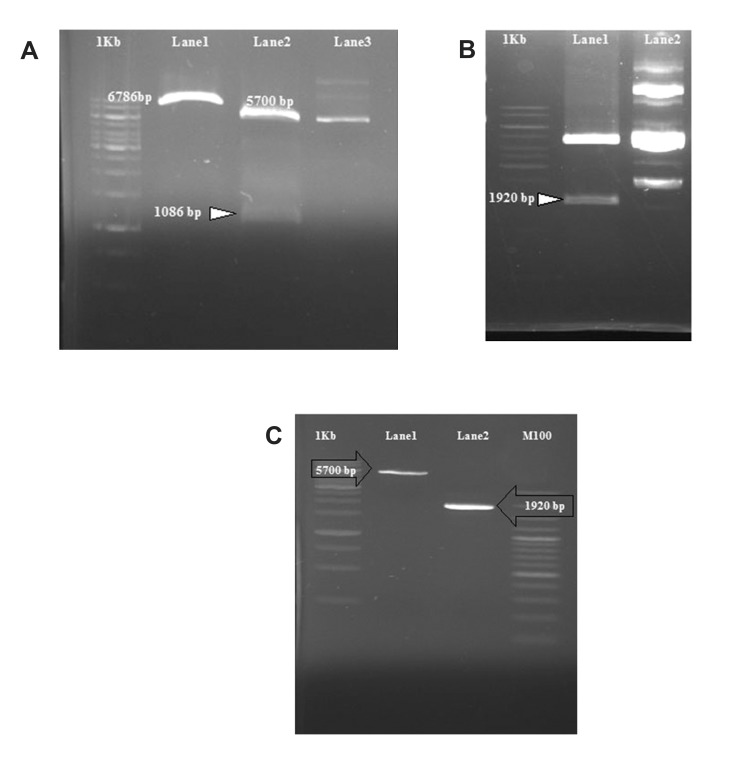
The pattern of restriction enzyme digestion on pET15b and pCMVInt A: Single digestion on pET15b with BamHI (Lane 1), double digestion on pET15b with NheI & BamHI (Lane 2) and undigested pET15b (Lane 3). B: double digestion on pCMVInt with KpaI & BamHI (Lane 1) and undigested pCMVInt (Lane 2). C: linearized pET15b plasmid (Lane 1) and phiC31 integrase open reading frame (Lane 2) after gel extraction.

Plasmids pCMVInt and pBCPB^+^ (Fig 1) containing phiC31 cDNA (GenBank accession no. X59938) and *att* site sequences, respectively, were gifts from Prof. M.P. Calos (Stanford University, USA) (3). Integrase-expressing plasmid was constructed as follows: pCMVInt vector was digested by *Kpn*I, and then was blunted using klenow fragment (Fermentas, Lithuania). In the second step, *Bam*HI excised the cDNA of phiC31 from pCMInt. At the same time, pET15b plasmid was digested by *Nde*I. Then, it was blunted by klenow fragment, and digested with *Bam*HI. pET15b (Novagen, CA, USA), backbone and integrase open reading frame, were extracted from the gel and ligated using DNA Ligation Kit (TaKaRa, Japan) (Fig 2). The recombinant pET-Int expression plasmid was amplified in a DH5α strain of E. coli (Invitrogen, USA)and sequenced with specific primer.

### Expression and purification of phiC31 integrase

*E.coli* BL21 (DE3) was transformed with 6xHis tagged phiC31 cDNA in pET15b, under induction with 1mM IPTG (Thermo Scientific, USA), and resulted protein was purified under non-denaturing condition using Nickel Affinity Gel Nickel Affinity Gel (Sigma, USA) according to manufacturer’s protocol. Protein concentration was assessed by Bradford method (15). Purified protein was concentrated by Amiconultrafiltration15 (Millipore, MA, USA). For evaluation of protein expression, the sodium dodecyl sulfate-polyacrylamide gel electrophoresis (SDS-PAGE) carried out.

The purified protein was freezed using submerging the samples in liquid nitrogen and rapidly was stored at -80℃. For maintenance of enzyme activity during the freezing process, the purified protein was preserved in 50% glycerol.

### *in vitro* evaluation of phiC31 integrase activity

For *in vitro* functionality assessment of phiC31 integrase, pBCPB^+^ plasmid (Fig 1) was used as an intra-molecular assay vector. pBCPB^+^ plasmid was incubated with crude lysate of transformed E. coli. *in vitro* recombination reactions included 1 µg of pBCPB^+^, 1 µg of crude lysate containing integrase, 20 mM Tris-HCl (pH= 7.5), 100 mM NaCl, 0.1 mM EDTA, and 1% glycerol in a final volume of 20 ml. Reactions were carried out at 37℃ for 1 hour, and then heat inactivated at 80℃ for 20 minutes.

### PCR screening for *in vitro* site-specific ecombination

One µl of *in vitro* recombination reaction was used as template in PCR for detection of *in vitro* site-specific recombination. Each PCR reaction contained 5 pM forward (5´-GGCGAGAAAGGAAGGGAAGA- 3´) and reverse (5´-ATTAACCCTCACTAAAGGGA- 3´) primer, 10 units of Ex-Taq DNA polymerase, 10 mM Tris- HCl (pH=7.9), 50 mM KCl, 1.5 mM MgCl_2_, 200 µM dNTPs. Touch-down PCR was performed as follows: 94℃ for 4 minutes, 14 cycles of 94℃ for 30 seconds, 66℃ for 30 seconds which was decreased 0.3℃ per each cycle, gradually, 72℃ for 45 seconds, 27 cycles of 94℃ for 30 seconds, 62℃ for 30 seconds, and 72℃ for 45 seconds. Final extension of PCR was carried out at 72℃ for 7 minutes. PCR products were visualized on 2% agarose gel.

## Results

**Fig 3:**
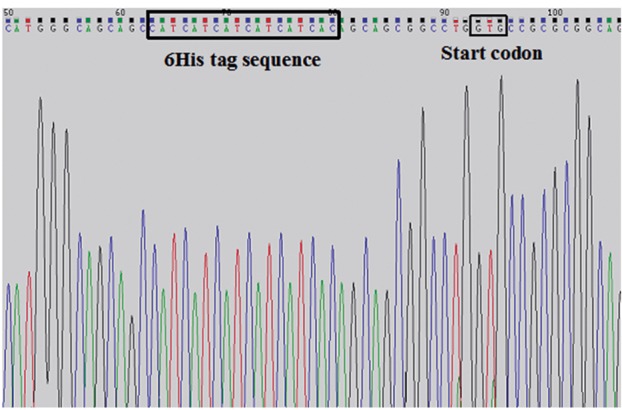
The results of partial phiC31 integrase cDNA sequencing.

The result of sequencing indicated that there was no mutation in phiC31 integrase cDNA sequence. On the other hand, the start codon (GTG) was placed in the same reading frame down-stream of 6xHis tag (Fig 3).

**Fig 4:**
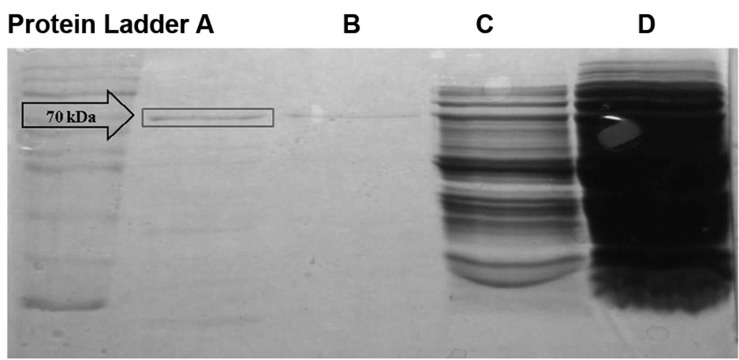
Expression of phiC31 integrase in Escherichia coli.

SDS-PAGE gel electrophosesis showed that the phiC31 integrase was expressed appropriately with the same expected size (70 kDa) in *E.coli* (Fig 4). Upon purification step of integrase, its concentration was determined by the Bradford assay method, which was 0.75 µg/µl.

**Fig 5:**
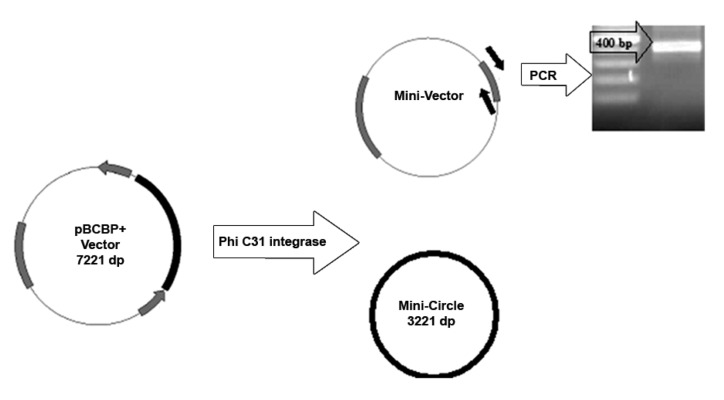
Schematic manner of *in vitro* phic31 integrase activity on intra-molecular assay vector. pBCPB^+^ contains attP and attB sites which are flanked of *lacZ* encoding sequence specific for intra-malecular assay. Functional phiC31 integrase creates two mini-circles according to att sites orientation on pBCPB^+^. A 400-bp product is indicative of site-specific recombination.

**Fig 6:**
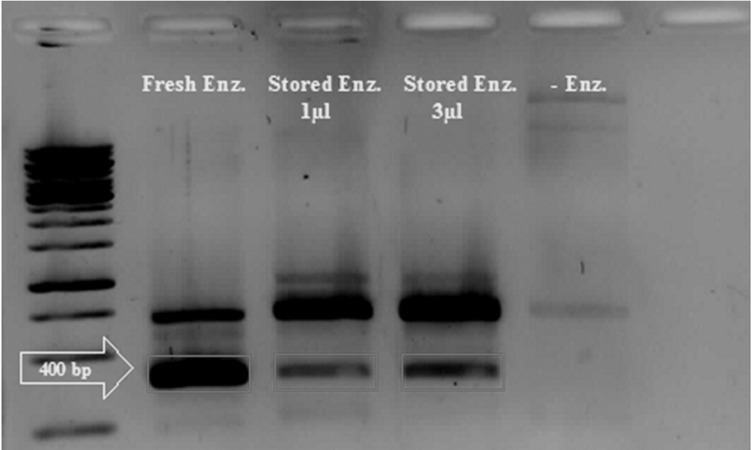
*in vitro* assessment of phiC31 integrase activity

*in vitro* phiC31 activity was confirmed using intra-molecular assay vector (pBCPB^+^) both by fresh and frozen-thawed protein. As the molecular assay vector contains both *attP* and *attB* sites in appropriate orientations (Fig 1) and intervening sequences. Having proper phiC31 integrase activity, recombination occurs in the vector of the specific DNA fragment located between *attP* and *attB*. This deletion could be verified easily by PCR (Fig 5). Data was evident that when the pBCPB^+^ assay plasmid was incubated with either fresh or frozen-thawed types of integrase, site-specific recombination was performed in the expected manner *in vitro* generating a 400 bp product, which was amplified in a PCR reaction (Fig 6).

## Discussion

Nowadays phiC31 integrase plays a powerful role in genetic engineering particularity in the field of stem cells and transgenic animals. Due to some unique features of phiC31 integrase including unidirectional activity and appropriate functionality without any co-factors requirement, this enzyme has been widely used in gene therapy and gene targeting (4). In our previous study, we utilized this system for creation of a stable Chinese hamster ovary (CHO) cell line expressing secretory type of tenecteplase protein (16). In this study, we tried to produce a recombinant type of phiC31integrase protein in *E.coli*. The SDS-PAGE analysis showed that phiC31 integrase was appropriately expressed with the expected size (70 kDa) (Fig 4). Notably, the extracted protein maintaned its activity after purification. In order to test the functionality of the phiC31 integrase, pBCPB^+^ was used as an intra-molecular assay vector (Fig 1). Especially, the presence of four amino acid residues (Ser-Ser-Gly-Leu) between 6xHis-tag and transcription start site of phiC31 integrase did not hamper enzyme activity as expected. Due to the presence *lacZ* sequence between two *att* sites orientation, the functional phiC31 integrase enzyme was able to delete DNA fragment encoding *lacZ* which created two mini-circles (Fig 5). Amplifying 400 bp product in PCR reaction indicated that the recombinant integrase protein had site-specific recombination activity (Fig 6). Similar approach for evaluation of phiC31 integrase activity reported in previous studies which have shown co-transfection of pCMVInt and pBCPB^+^ plasmids into human and bovine cells, caused generation of a specific 400 bp as an indicator for phiC31 activity (3, 6, 17). PCR screening for site-specific integration showed that the phiC31 integrase was functional even after snap-freezing despite the slight decrease in enzyme activity (Fig 6). It seems that deep freeze storage of recombinant phiC31 integrase have negative effect on its activity (Fig 6). In this regards, recently, McEwan et al. (18) have proposed a buffer containing Zn^2+^ as an essential factor for high-affinity DNA binding and recombinase activity of phiC31 integrase. Applying this buffer may improve *in vitro* phiC31 activity after freezing which needs to be investigated in future studies.

## Conclusion

The results showed that the produced recombinant protein has reasonable functionality *in vitro*. Thus it could be useful to be used in our next studies including protein microinjection in oocyte in the aim of creation of transgenic animal or gene therapy.
